# Droplet Digital Polymerase Chain Reaction Assay for Quantifying *Salmonella* in Meat Samples

**DOI:** 10.3390/foods15020337

**Published:** 2026-01-16

**Authors:** Yingying Liang, Yangtai Liu, Xin Liu, Jin Ding, Tianqi Shi, Qingli Dong, Min Chen, Huanyu Wu, Hongzhi Zhang

**Affiliations:** 1 Shanghai Municipal Center for Disease Control and Prevention, Shanghai 200336, China; liangyingying@scdc.sh.cn (Y.L.); shitianqi@scdc.sh.cn (T.S.); chenmin@scdc.sh.cn (M.C.); 2 School of Health Science and Engineering, University of Shanghai for Science and Technology, Shanghai 200093, China; usstlyt@163.com (Y.L.); xinliu0415@163.com (X.L.); dongqingli@126.com (Q.D.); 3 Shanghai Pudong New Area Center for Disease Control and Prevention, Shanghai Pudong New Area Health Supervision Institute, Shanghai 200136, China; dj951130@163.com

**Keywords:** ddPCR, *Salmonella*, quantitative detection, specificity, sensitivity

## Abstract

*Salmonella*, a major global foodborne pathogen, is a leading cause of salmonellosis. Quantitative detection of *Salmonella* provides a scientific basis for establishing microbiological criteria and conducting risk assessments. The plate count method remains the primary approach for bacterial quantification, whereas the most probable number (MPN) method is commonly used for detecting low levels of bacterial contamination. However, both methods are time-consuming and labor-intensive. Validated digital polymerase chain reaction (dPCR) techniques are emerging as promising alternatives because they enable rapid, absolute quantification with high specificity and sensitivity. Herein, we developed a novel droplet dPCR (ddPCR) assay for identifying and quantifying *Salmonella* using *invA* as the target. The assay demonstrated high specificity and sensitivity, with a limit of quantification of 1.1 × 10^2^ colony-forming units/mL in meat samples. Furthermore, the log_10_ values obtained via ddPCR and plate counting exhibited a strong linear relationship (R^2^ > 0.99). Mathematical modeling of growth kinetics further confirmed a high correlation between plate count and ddPCR measurements (Pearson correlation coefficient: 0.996; calculated bias factor: 0.88). Collectively, these results indicate that ddPCR is a viable alternative to the MPN method and represents a powerful tool for the quantitative risk assessment of food safety.

## 1. Introduction

*Salmonella* is the causative agent of salmonellosis. Recent data from China’s foodborne disease surveillance program highlight the substantial public health burden posed by foodborne pathogens, with *Salmonella* identified as the leading causative microbial agent. Meat and meat products are among the primary sources of foodborne disease outbreaks [1]. Therefore, the development of an efficient and accurate method for the quantitative detection of viable *Salmonella* is critical.

Quantitative detection of pathogenic microorganisms in food provides a scientific basis for the implementation of microbiological criteria and the facilitation of risk assessments [2,3]. According to a 2004 technical risk assessment of *Salmonella* in eggs and broilers conducted by the Food and Agriculture Organization (FAO) of the United Nations and the World Health Organization (WHO) [4], reductions in the prevalence of *Salmonella* in poultry flocks lead to a directly proportional decrease in risks to human health. Adherence to food microbiological criteria is therefore essential for protecting consumer health. China and other major trading countries apply different microbiological regulatory limits for foodborne pathogens. Major international organizations, including the FAO and WHO, have established stringent microbiological criteria for *Salmonella* in dairy products, meat products, eggs, and egg products. Consequently, the development of strict, scientifically grounded regulatory limits for *Salmonella* in foods produced in China would substantially enhance their competitiveness in the global market while ensuring food safety and safeguarding public health.

Current methods for quantifying viable bacteria still primarily rely on culture-based approaches. The gold standard is the plate count method, which is recommended and widely adopted for microbiological enumeration by leading international organizations, including the FAO, WHO, International Commission on Microbiological Specifications for Foods, and International Organization for Standardization [5]. Another culture-based approach is the most probable number (MPN) technique, which is commonly applied to samples with low bacterial loads [6]. The Food and Drug Administration currently recommends the MPN method for quantifying *Salmonella* in food [7]. However, as an indirect method, its reliability depends on the characteristics of the food matrix [6]. Additionally, both plate count and MPN methods are time-consuming and labor-intensive. An alternative approach is quantitative polymerase chain reaction (qPCR), which has been widely used to detect and quantify *Salmonella* and other foodborne pathogens [8,9]. However, qPCR is typically regarded as a relative quantification method because it relies on standard curves and quantification cycle values [10,11].

Advances in PCR technology and instrumentation have led to the development of droplet digital PCR (ddPCR). This third-generation nucleic acid amplification technology partitions a single reaction into thousands to millions of nanoliter-sized droplets (typically ranging from 10^3^ to 10^7^, depending on the platform), each functioning as an independent PCR reaction. The fraction of positive droplets is then used to calculate target concentrations based on Poisson statistics. Consequently, ddPCR enables absolute quantification of target nucleic acids (copies/µL) without the need for internal reference genes or standard curves [12,13,14,15]. ddPCR has been widely used for detecting various pathogenic bacteria, including *Vibrio parahaemolyticus*, *Pseudomonas aeruginosa*, *Pseudomonas fragi*, *Salmonella*, and *Shigella*. Studies have demonstrated that ddPCR exhibits high sensitivity, good reproducibility, and strong potential for operational standardization [16,17,18,19]. Moreover, as an endpoint assay, ddPCR shows high tolerance to PCR inhibitors, thereby minimizing bias introduced by complex food matrices. However, most previous studies have primarily emphasized the methodological advantages of ddPCR, such as sensitivity and specificity, without systematically evaluating the accuracy of its quantitative performance. Conversely, the present study focuses on assessing the consistency between ddPCR and plate count results, with the aim of providing empirical evidence to support the application of ddPCR for the quantitative detection of *Salmonella*.

In this study, we developed a ddPCR assay for the quantitative detection of *Salmonella* and evaluated its performance by (i) assessing key analytical parameters, including specificity, sensitivity, and selectivity; (ii) comparing growth parameters derived from ddPCR with those obtained using plate count methods through application of the Baranyi–Roberts growth model; and (iii) comparing the performance of ddPCR with that of the MPN method using real food samples.

## 2. Materials and Methods

### 2.1. Bacterial Strains

A total of 32 common *Salmonella* serotype isolates and 8 non-*Salmonella* isolates were included in this study (Table 1). *Salmonella typhimurium* (ATCC 14028) [20] was used for ddPCR assay development and evaluation and for predictive growth modeling. The non-*Salmonella* strains comprised *Escherichia coli* O157:H7, *Yersinia enterocolitica*, *Klebsiella pneumoniae*, and *Cronobacter sakazakii* (formerly *Enterobacter sakazakii*), along with the Gram-positive bacteria *Staphylococcus aureus*, *Listeria monocytogenes*, *Clostridium perfringens*, and *Bacillus cereus*. Non-reference isolates were identified in our laboratory using a VITEK automated microbial biochemical identification system (bioMérieux, Marcy-l’Étoile, France).

### 2.2. Primers and Probes

*invA* (GenBank accession no. MK017942.1), which encodes invasion protein A, was selected as the amplification target. This gene has been reported to be specific to 126 *Salmonella* serovars [21]. Primers and probes were designed using Primer Premier 5.0 software (Premier Biosoft, Toronto, ON, Canada) and synthesized by Sangon Biotech (Shanghai, China). The sequences of the primers and probe were as follows: *invA*-F (5′-AGCGTACTGGAAAGGGAAAG-3′), *invA*-R (5′-CACCGAAATACCGCCAATAAAG-3′), and *invA*-P (5′-FAM-TACGGTTCCTTTGACGGTGC-BHQ1-3′).

### 2.3. ddPCR

Bacterial strains were cultured overnight in nutrient broth at 37 °C. On the following day, 1 mL of each saturated culture was serially diluted 10-fold in phosphate-buffered saline (pH 7.4) and subjected to DNA extraction. In parallel, bacterial concentrations in the saturated cultures were determined using the plate count method in accordance with the GB 4789.4 standard [21]. Genomic DNA was extracted using a QIAamp DNA Mini Kit (Qiagen, Germantown, MD, USA) according to the manufacturer’s instructions, with minor modifications. Briefly, bacterial suspensions were centrifuged, the resulting pellets were resuspended in deionized water and recentrifuged, and the pellets were then processed for DNA extraction following the standard protocol.

The TargetingOne ddPCR system (TargetingOne Corp., Beijing, China) was used for droplet generation, amplification, and data analysis according to the manufacturer’s instructions. Briefly, each 30-µL PCR reaction mixture contained 5 µL of 4× Probe dPCR Unimix (TargetingOne Corp.), 300 nM each of forward and reverse primers, 150 nM of probe, 2 µL of DNA template, and nuclease-free water to volume. The reaction mixture was loaded onto a ready-to-use disposable plastic chip and combined with 160 µL of droplet generation oil. The chip was then inserted into a droplet maker (TargetingOne Corp.) for droplet generation. Generated droplets were subjected to PCR amplification under the following thermal cycling conditions: initial denaturation at 95 °C for 10 min; 40 cycles at 94 °C for 30 s and 60 °C for 1 min (annealing/extension); followed by 12 °C for 5 min and an infinite hold at 4 °C. A ramp rate of 1.5 °C/s was applied for all steps. After amplification, droplets were read using a chip reader (TargetingOne Corp.), and copy numbers were calculated based on Poisson statistics. Data were exported to a spreadsheet for concentration calculations. Samples with confidence limits governed by a Poisson distribution were further analyzed using Chip Reader R1 software (TargetingOne Corp.).

Optimal PCR performance was assessed based on the following criteria: clear separation between positive and negative droplet populations, a high copy number detection rate, and minimal “rain,” defined as droplets occurring between the positive and negative droplet clusters.

### 2.4. Specificity and Selectivity Analyses

Following overnight culture of the bacterial strains listed in Table 1, 1 mL of each culture was used for DNA extraction and subsequent evaluation of ddPCR specificity. To assess ddPCR selectivity, *Salmonella* isolates at concentrations below 10^3^ colony-forming units (CFU)/mL were mixed with eight non-*Salmonella* isolates at concentrations exceeding 10^6^ CFU/mL before analysis.

### 2.5. Sensitivity Analysis

**Figure 1:**
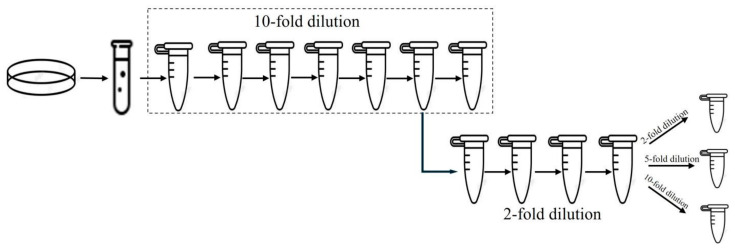
Serial dilution scheme used to determine the LOQ of the ddPCR assay.

The limit of quantification (LOQ) refers to the lowest concentration in a sample that an analytical method can reliably and quantitatively determine. To determine the LOQ, meat samples (25 g of fresh pork) were artificially contaminated via aseptic inoculation with a 1-mL aliquot of a pure overnight bacterial culture. After homogenization, the samples were subjected to a 10-fold serial dilution to obtain a dynamic concentration range, followed by a 2-fold dilution series spanning additional gradients. To determine the LOQ of ddPCR, the low-concentration samples were subjected to 2-, 5-, and 10-fold dilutions, aiming to achieve the lowest possible concentration levels (Figure 1). For each low concentration level, 10 independent replicate tests were conducted. The relative standard deviation (RSD) was calculated using the independent replicate test results to assess the precision of the ddPCR assay for quantitative detection. ddPCR accuracy was evaluated by calculating the recovery rate for each concentration.

For ddPCR analysis, linearity across the dynamic range was evaluated using the correlation coefficient (R^2^), calculated from the mean copy numbers obtained from replicate dilution series. Copy numbers generated by ddPCR were converted to bacterial concentrations using Equation (1):

(1) $$C\;=\;\frac{N\;\times V1}{V2}$$

where *C* represents the bacterial concentration (CFU/mL), *N* is the copy number (copies/µL), *V*_1_ is the final volume of extracted nucleic acid (µL), and *V*_2_ is the volume of the extracted bacterial suspension (mL).

### 2.6. Detection of Salmonella in Meat Samples Using the MPN Method and ddPCR

Between April and August 2024, 150 meat samples were purchased from supermarkets and farmers’ markets in Shanghai, including pork (*n* = 61), beef (*n* = 51), mutton (*n* = 9), and poultry (*n* = 29). Each sample was weighed, labeled, placed in an individual sterile bag, and transported to the laboratory under refrigerated conditions within 30 min for immediate analysis.

*Salmonella* loads in the samples were quantified using both the MPN method and ddPCR. For MPN determination, the three-tube MPN method was performed according to the *Workbook on National Monitoring of Pollutants and Harmful Factors*. Briefly, 300 g of each meat sample was homogenized with 300 mL of buffered peptone water (BPW). A 1-mL aliquot of the homogenate was inoculated into 9 mL of BPW in each of three tubes. The inoculated tubes were incubated at 37 °C for 18 h, after which 1-mL aliquots were transferred to 10 mL of tetrathionate brilliant green broth and incubated at 42 °C for 24 h. Following enrichment, cultures were streaked onto selective agar media, including xylose lysine deoxycholate agar plates and chromogenic *Salmonella* agar plates (Shenqi Co., Shanghai, China), and incubated at 37 °C for 24 h. Presumptive *Salmonella* colonies were identified using standard biochemical tests [20]. The MPN values were calculated based on the number of positive tubes in each of the three dilution sets using standard MPN tables.

For ddPCR analysis, DNA was extracted from 10 mL of each sample homogenate as described in Section 2.3. Each sample was analyzed in duplicate.

### 2.7. Mathematical Modeling of S. typhimurium Growth Kinetics

For growth kinetics modeling, *S. typhimurium* was inoculated into nutrient broth at an initial concentration of 2 log_10_ CFU/mL and incubated at 37 °C. Bacterial concentrations were determined at hourly intervals using both ddPCR and the plate count method, based on preliminary experiments. Each time point was analyzed in triplicate. Before modeling, ddPCR data (copies per 20 µL reaction) were converted to logarithmic bacterial concentrations (log_10_ CFU/mL) using Equation (2) [22]:

(2) $$Y_{ddPCR}=\log_{10}\left(N\times \frac{V_{1}}{V_{2}{\times V}_{3}}\right)$$

where $Y_{ddPCR}$ is the logarithm of the bacterial concentration (log_10_ CFU/mL), N is the nucleic acid copy number detected in the 20-µL ddPCR reaction (copies/20 µL), $V_{1}$ is the final volume of extracted nucleic acids (µL), $V_{2}$ is the volume of nucleic acid template added to the ddPCR reaction (µL), and $V_{3}$ is the volume of the extracted bacterial suspension (mL). The values of $V_{1}$, $V_{2}$, and $V_{3}$ were 50 µL, 1 µL, and 1 mL, respectively.

Pearson correlation analysis and the bias factor (BF) were used to compare the relative accuracy of ddPCR and the plate count method. A BF > 1 indicates a fail-dangerous situation, whereas a BF < 1 indicates a fail-safe situation [23]. The BF was calculated using Equation (3):

(3) $$BF=10^{\frac{1}{n}\sum_{i=1}^{n}\log_{10}\left(Y_{Platei}{/Y}_{ddPCRi}\right)}$$

where n is the number of observation points, and $Y_{Platei}$ and $Y_{ddPCRi}$ are the bacterial concentrations determined using the plate count method and ddPCR, respectively (log_10_ CFU/mL). The acceptable prediction zone was also evaluated [24], with values ≥ 70% considered indicative that ddPCR is an acceptable alternative to the plate count method for bacterial quantification.

The complete Baranyi–Roberts growth model (Baranyi and Roberts, 1994) [25] was applied to estimate bacterial growth kinetic parameters (Equation (4)):

(4) {Y=Y0+A(t)ln10−log10{1+exp[A(t)]−110^(Ymax−Y0)}A(t)=μmaxt+ln[exp(−μmaxt)+exp(−μmaxλ)−exp(−μmaxt−μmaxλ)]

where *Y* is the logarithm of the bacterial concentration at time t (log_10_ CFU/mL), $Y_{0}$ and $Y_{max}$ are the logarithms of the initial and maximum bacterial population densities (log_10_ CFU/mL), respectively, $\mu_{max}$ is the maximum specific growth rate (h^−1^), and λ is the lag time (h).

The goodness-of-fit of the model was evaluated using the root mean square error (RMSE), calculated as shown in Equation (5) [26]:

(5) $$RMSE=\sqrt{\frac{\sum_{i=1}^{n}{\left(Y_{i}-\hat{Y_{i}}\right)}^{2}}{n}}$$

where *n* is the number of observation points, $\hat{Y_{i}}$ is the model-fitted bacterial concentration (log_10_ CFU/mL), and $Y_{i}$ is the experimentally determined bacterial concentration obtained via either the plate count method or ddPCR (log_10_ CFU/mL).

### 2.8. ddPCR Data Analysis

Thresholds for each ddPCR experiment were manually defined based on the following criteria. First, positive and negative droplet clusters were verified to be completely separated using fluorescence amplitude plots. The threshold was then placed at the clear minimum in fluorescence signal intensity between the two clusters, ensuring that it exceeded the fluorescence intensity of all droplets in the negative control samples. All experiments were performed in duplicate to verify data reproducibility. Repeatability was evaluated by calculating the coefficient of variation in the measured values obtained from ddPCR measurements within a single experimental run.

## 3. Results

### 3.1. Primer Pair and Probe Specificity

All 32 *Salmonella* isolates tested positive for *invA*, whereas all 8 non-*Salmonella* isolates tested negative (*invA* copy number = 0 copies/µL; Table 1). These results demonstrate that the primer pair and probe exhibit high specificity for *Salmonella*.

### 3.2. ddPCR Selectivity and Sensitivity

To evaluate the selectivity of ddPCR for detecting the *Salmonella invA* gene, seven non-*Salmonella* isolates at high concentrations (>10^6^ CFU/mL) were used as background interference. The ddPCR assay reliably detected low concentrations of *S. typhimurium* (<10^3^ CFU/mL) even in the presence of high background bacterial loads (Table 1). ddPCR yielded 45.1 copies/µL for pure *S. typhimurium* cultures and 42.5–46.8 copies/µL for *S. typhimurium* in mixed cultures containing background interference, demonstrating high consistency across sample types. The RSD between measurements obtained from pure and mixed cultures ranged from 0.93% to 4.20%, indicating excellent selectivity of the ddPCR assay for detection of *S. typhimurium invA*.

**Figure 2:**
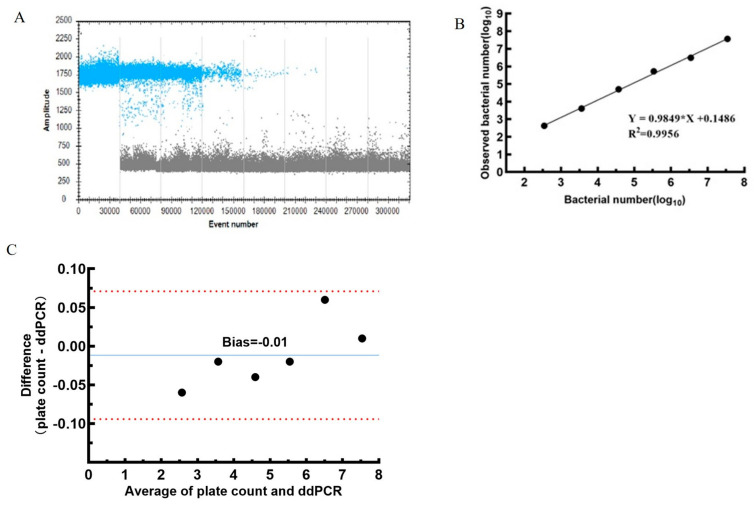
Comparison of ddPCR results and plate count results. (**A**) The ddPCR fluorescence amplitude plots. Blue and grey represents positive and negative droplet populations, respectively. (**B**) Linearity of ddPCR quantification across the dynamic range, assessed by the correlation coefficient (R^2^) calculated from mean copy numbers obtained from serially diluted samples. (**C**) Comparison of results of ddPCR and plate count using Bland-Altman.

The detection and quantification performance of ddPCR was evaluated using 10-fold serial dilutions of *S. typhimurium* DNA extracted from corresponding 10-fold serial dilutions of bacterial cultures, starting from an initial concentration of 3.6 × 10^7^ CFU/mL (Table 2 and Table 3). The ddPCR fluorescence amplitude plots demonstrated clear separation between positive and negative droplet populations (Figure 2A). Regression analysis of ddPCR and plate count data yielded the equation *y* = 0.9849*x* + 0.1486, with an R^2^ value of 0.9956, indicating an exceptionally strong linear correlation between the two methods (Figure 2B). The regression slope was close to unity (0.9849), and the intercept was minimal (0.1486). The maximum RSD observed across the tested concentration range (3.6 × 10^7^–3.6 × 10^2^ CFU/mL) was 20.2%, demonstrating consistent quantitative performance. Collectively, these results further confirm the reliability of ddPCR for absolute quantification of *Salmonella*.

As the bacterial concentration decreased, the RSD between plate count and ddPCR results increased. When the dilution reached a theoretical concentration of 1.1 × 10^2^ CFU/mL, the plate count yielded 18 CFU/mL, corresponding to a recovery rate of 16.3%, which was lower than expected (Table 2). At the same concentration, ddPCR measurements from 10 independent replicates exhibited an RSD of 14.2% and recovery rates ranging within 80–129%, indicating that both the precision and accuracy of ddPCR were acceptable and reproducible at 1.1 × 10^2^ CFU/mL. However, further dilution (5- and 10-fold) resulted in markedly increased RSD values of 52.2% and 133%, respectively, along with substantially broadened recovery ranges of 63.6–205% and 0–386%, respectively (Table 3). These results indicate that the precision and accuracy of ddPCR become unreliable at bacterial concentrations below 1.1 × 10^2^ CFU/mL.

### 3.3. Salmonella Detection in Meat Samples

Among the 150 meat samples analyzed using both the MPN method and ddPCR, 118 samples (78.7%) yielded concordant results between the two methods. Specifically, 107 samples were consistently negative for *Salmonella* (MPN method: <0.03 CFU/mL; ddPCR: 0 copies/µL), and 11 samples were consistently positive (MPN method: 0.036 to >11.000 CFU/mL; ddPCR: 14.0–307.6 copies/µL). The remaining 32 samples (21.3%) showed discrepant results. Of these, 26 samples tested negative by the MPN method but positive by ddPCR, with *Salmonella invA* detected at concentrations ranging within 12.2–339.8 copies/µL. Additionally, 6 samples could not be quantified using the MPN method, whereas ddPCR detected *Salmonella* at levels of 12.8–144.8 copies/µL in these samples (Table 4). Overall, these findings highlight the superior sensitivity and accuracy of ddPCR in detecting *Salmonella* in meat samples, particularly at low contamination levels.

### 3.4. Comparison of S. typhimurium Growth Kinetics Determined Using ddPCR and the Plate Count Method

The plate count results were highly correlated with the ddPCR results (Pearson correlation coefficient: 0.996). The calculated BF was 0.88, indicating a fail-safe situation. Meanwhile, 21/23 (91.3%) of the observation residuals ($Y_{Platei\;}{-\;\;Y}_{ddPCRi}$) were within the acceptable prediction zone of −1 log –0.5 log [24] (Table 5). These findings indicate that ddPCR is an acceptable alternative method for *Salmonella* quantification.

**Figure 3:**
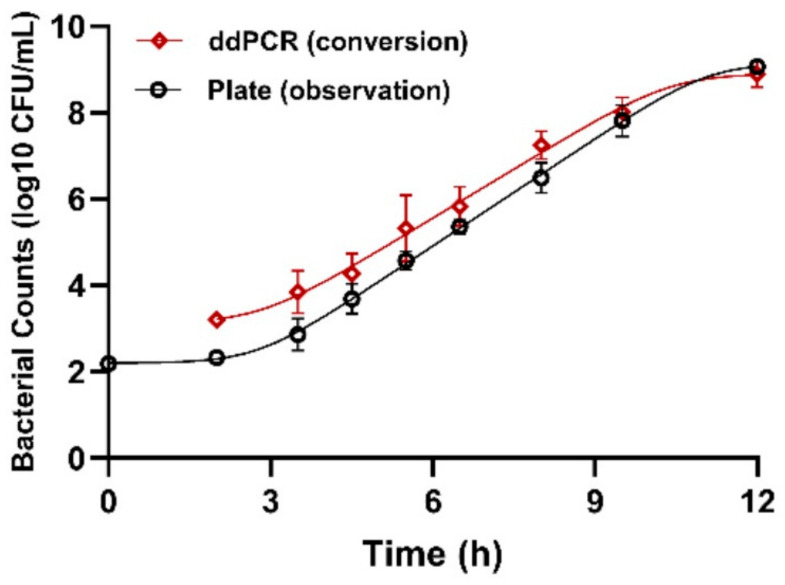
Growth curve fitting of *S. typhimurium* at 25 °C based on the plate count method and ddPCR assay.

*Salmonella* exhibited a typical sigmoid growth pattern at 25 °C, as demonstrated by both the traditional plate count method and ddPCR (Figure 3). The complete Baranyi–Roberts model was applied to describe the growth kinetics, and both methods showed similar and acceptable goodness-of-fit, with RMSE values below 0.5 log_10_ CFU/g (Table 5). Notably, the initial population density $Y_{0}$ estimated using ddPCR corresponded closely to the observation at the second sampling time point. The maximum specific growth rate $\mu_{max}$ estimated using the plate count method was slightly higher, whereas estimates of lag time λ and $Y_{max}$ were comparable between the two methods.

## 4. Discussion

ddPCR has been widely used for detecting foodborne pathogens [16,17,18,19], demonstrating that molecular quantification of specific target genes in food samples can provide a rapid, straightforward, and cost-effective means of assessing food quality and safety. In the present study, we established a ddPCR assay for the quantitative detection of *Salmonella* in food.

Results from 10 independent replicate assays showed that the amplification copy number in the 20-µL ddPCR reaction ranged within 3.51–5.67 copies, with an RSD of 14.2% and a mean bacterial concentration of 1.1 × 10^2^ CFU/g. These findings confirm that the LOQ of the ddPCR assay developed in this study is 1.1 × 10^2^ CFU/g, which is consistent with previous reports demonstrating ddPCR sensitivities as low as 10^2^ CFU/g [27,28,29]. Moreover, the results indicate that ddPCR can detect as few as three target copies; however, measurements below this threshold should be considered unreliable for quantitative analysis.

The MPN method is commonly used to quantify low levels of *Salmonella* in food products [30]. Given the typically low levels of *Salmonella* contamination in meat and the potential interference from background microbiota, this study compared results obtained using ddPCR with those obtained using the MPN method rather than the plate count method. Comparison of *Salmonella* detection in 150 meat samples revealed an overall concordance of 78.6% (118/150 samples) between ddPCR and MPN. One explanation for the observed discrepancies lies in the inherent limitations of the MPN approach. In several cases, *Salmonella* was detected in higher-dilution samples but not in lower-dilution samples from the same specimen. This non-monotonic detection pattern deviates from the assumptions underlying the MPN method and prevents reliable quantification using standard MPN reference tables.

Another potential reason for the observed inconsistency is that the MPN method yielded negative results in cases where ddPCR was able to quantitatively detect *Salmonella*. The underlying causes of this discrepancy are complex and may be related to the physiological state of *Salmonella* in meat samples. First, ddPCR is capable of detecting DNA from dead *Salmonella* cells, whereas the MPN method detects only viable, culturable bacteria. Second, *Salmonella* may exist in a viable but nonculturable (VBNC) state in meat samples, in which cells cannot be recovered using culture-based methods but can still be detected and quantified via ddPCR, as reported in multiple studies. For example, Ou et al. [31] reported the presence of VBNC *Salmonella* isolates in food, and Zhao et al. and Lv et al. [32,33] developed propidium monoazide–ddPCR assays for the detection of VBNC *V. parahaemolyticus* and VBNC *C. sakazakii*, respectively, demonstrating efficient detection of low-abundance VBNC pathogens in food matrices. Based on these findings, further investigation is warranted to clarify the impact of dead bacterial cells on ddPCR-based detection. Importantly, the ability of ddPCR to detect VBNC bacteria overcomes a key limitation of the MPN method and has significant implications for food safety monitoring and risk assessment.

The CFU values converted from ddPCR measurements showed a consistent correlation with observed CFU counts obtained by plate counting. Model-fitting results further indicated that these converted CFU values were effective for estimating *Salmonella* growth kinetics. Notably, the converted CFU values were initially higher than the corresponding plate count results, but this discrepancy gradually decreased as bacterial growth progressed. This pattern suggests the presence of VBNC bacteria in the meat samples. Although VBNC cells cannot grow and form colonies on conventional culture media, they remain viable and metabolically active [34,35] and can therefore be detected by ddPCR. Importantly, despite their inability to form colonies on agar plates, VBNC bacteria retain the capacity to resuscitate in the human intestinal tract, thereby posing latent health risks [36]. Accordingly, ddPCR-based quantification has the potential to improve the accuracy of predictive microbiological modeling, particularly in situations where unculturable cells are present. Moreover, these findings highlight a potential link between molecular quantification methods and traditional culture-based assessments, contributing to a more integrated understanding of microbial population dynamics.

In this study, ddPCR detection results were converted into CFU values. Although this conversion relies on the theoretical assumptions that *Salmonella* is a unicellular organism and that the *invA* target gene is present as a single copy per cell, the feasibility of this approach was experimentally validated. Specifically, CFU values of bacterial suspensions were first determined via plate counting. The suspensions were then serially diluted, and total DNA was extracted from each dilution. ddPCR analysis and plate counting were performed in parallel. Copy numbers obtained via ddPCR were converted to CFU values, and linear regression analysis was conducted between the two datasets to generate a standard curve (Figure 2). The regression analysis yielded an R^2^ of 0.9956, with a slope of 0.9849 and an intercept of 0.1486, indicating a high level of agreement between ddPCR-derived and plate count–derived results. Consistent with our findings, Oliver et al. reported strong agreement between ddPCR detection of *L. monocytogenes* and plate count results, with relative deviations of <30% [34]. Nevertheless, it should be noted that although ddPCR results correlate well with plate count data, ddPCR quantifies total *Salmonella* DNA and does not directly reflect the number of viable cells. Consequently, ddPCR cannot currently replace the plate count method as the primary basis for establishing food microbiological limit standards. Instead, ddPCR represents a powerful complementary tool for applications such as quantitative microbial risk assessment, method development, and investigations of low-level food contamination.

One of the main limitations of ddPCR is its relatively narrow linear dynamic range (approximately 1.1 × 10^2^–3.6 × 10^7^ CFU/µL in this study), making the selection of an appropriate dilution factor critical for accurate quantification. For example, in *Campylobacter* detection, ddPCR signal saturation has been reported at concentrations as low as 500 pg/µL [37]. In the present study, no signal saturation was observed during ddPCR analysis of the 150 meat samples, likely due to the generally low levels of *Salmonella* contamination (maximum of 339 copies per 20 µL reaction). Nevertheless, the applicability of ddPCR should be further validated using larger and more diverse sample sets, with particular attention to food matrices characterized by low-level contamination.

## 5. Conclusions

This study established a ddPCR assay for the identification and quantitative detection of *Salmonella* in food using *invA* as the target. The LOQ of the assay was determined to be 1.1 × 10^2^ CFU/g. To our knowledge, this is the first method to achieve quantitative results with an RSD below 25% when compared with the plate count method. Moreover, quantification results obtained using ddPCR showed a strong correlation with those derived from plate counts (Pearson correlation coefficient = 0.996; bias factor = 0.88). Collectively, these findings demonstrate that ddPCR is not only an acceptable alternative for *Salmonella* quantification but also a robust approach for improving the accuracy of absolute quantification. The application of this method can provide reliable scientific data to support food microbiological risk assessment and contribute to the strengthening of national food safety management.

## Figures and Tables

**Table 1:** Selectivity of the ddPCR assay in detecting *invA* in *Salmonella*.

Groups	Pure Culture	ddPCR (Copies/µL)	Bacterial Conbinations	ddPCR (Copies/µL)	RSD/%
1	S. *typhimurium*	45.1	*E. coli +* *S. typhimurium*	43.2 ± 0.64	3.04
2			*Y. enterocolitica +* *S. typhimurium*	46.1 ± 0.21	1.55
3			*K. pneumoniae + S. typhimurium*	46.8 ± 0.57	2.62
4			*E. sakazakii +* S. typhimurium	44.3 ± 0.49	1.27
5			*S. aureus +* S. typhimurium	45.7 ± 0.57	0.93
6			*L. monocytogenes +* S. typhimurium	43.9 ± 0.21	1.91
7			*B. cereus +* S. typhimurium	42.5 ± 0.35	4.20

Note: Data are presented as the mean ± standard deviation of two independent replicates. RSD, relative standard deviation.

**Table 2:** Comparison of ddPCR and plate count results obtained from 10-fold serial dilutions of pure cultures.

Expected CFU/g of Pure Culture	Results of Plate Counting (CFU/g)	Results of ddPCR		RSD/%
		Copies/Reaction	CFU/g (Converted Using Copies)	
3.6 × 10^7^	3.5 × 10^7^	670,910.8	3.4 × 10^7^	2.94%
3.6 × 10^6^	3.5 × 10^6^	60,060.4	3.0 × 10^6^	10.88%
3.6 × 10^5^	3.4 × 10^5^	7050.7	3.5 × 10^5^	2.05%
3.6 × 10^4^	3.7 × 10^4^	820.0	4.1 × 10^4^	7.25%
3.6 × 10^3^	3.6 × 10^3^	76.0	3.8 × 10^3^	3.82%
3.6 × 10^2^	3.5 × 10^2^	8.0	4.0 × 10^2^	9.43%
1.8 × 10^3^	1.7 × 10^3^	35.8	1.8 × 10^3^	4.04%
8.8 × 10^2^	8.8 × 10^2^	22.0	1.1 × 10^3^	15.17%
4.4 × 10^2^	4.3 × 10^2^	9.0	4.5 × 10^2^	3.21%
2.2 × 10^2^	1.8 × 10^2^	4.8	2.4 × 10^2^	20.2%
1.1 × 10^2^	18	-	-	-

Note: Samples were subjected to 10-fold serial dilution to generate a dynamic concentration range. RSD between results obtained using the plate count method and the ddPCR assay. “-” indicates that the corresponding result is presented in Table 3.

**Table 3:** ddPCR detection performance at low concentration levels.

2-Fold Dilution				5-Fold Dilution				10-Fold Dilution			
Copies/20 µL	CFU/g	RSD	Recovery Rate	Copies	CFU/g	RSD	Recovery Rate	Copies	CFU/g	RSD	Recovery Rate
5.67	1.4 × 10^2^	14.20%	80–129%	3.60	90	52.20%	63.6–205%	3.40	85	113.8%	0–386%
5.59	1.4 × 10^2^			3.49	87			1.27	32		
5.51	1.4 × 10^2^			3.48	87			1.16	29		
4.78	1.2 × 10^2^			3.32	83			1.12	28		
4.74	1.2 × 10^2^			2.34	59			1.07	27		
4.53	1.1 × 10^2^			1.19	30			1.07	27		
4.51	1.1 × 10^2^			1.18	29			0.00	0		
4.42	1.1 × 10^2^			1.17	29			0.00	0		
4.33	1.1 × 10^2^			1.17	29			0.00	0		
3.51	88			1.16	28			0.00	0		

Note: To determine the LOQ of ddPCR, low-concentration samples from Table 2 were further diluted 2-, 5-, and 10-fold to achieve progressively lower concentration levels. RSD values were calculated based on 10 independent replicates. Recovery rate represents the ratio of ddPCR-measured concentrations to the theoretical concentrations.

**Table 4:** Detection of *Salmonella* in 150 meat samples using the ddPCR assay and MPN method.

No. of Samples	MPN Results/g	ddPCR Results (Copies/g)	Result of McNemar Test
107	<0.03	0	χ2 = 24.04, *p* < 0.001
11	0.036–>11	700–15,380	
26	<0.03	610–16,990	
6	-a	640–7240	-

Note: ^a^ MPN results could not be determined using standard reference tables.

**Table 5:** Estimated growth kinetic parameters of *S. typhimurium* at 25 °C determined using the plate count method and ddPCR assay.

Parameter	Plate Counting (Observation)		ddPCR (Conversion)	
	Estimated Value	Standard Error	Estimated Value	Standard Error
*Y*_0_ (log_10_ CFU/g)	2.2	0.1	3.1	0.012
μ_max_ (1/h)	1.909	0.069	1.746	0.143
*λ* (h)	2.7	0.2	2.8	0.7
*Y_max_* (log_10_ CFU/g)	9.1	0.2	8.9	0.3
RMSE (log_10_ CFU/g)	0.221		0.397	

## Data Availability

The original contributions presented in this study are included in the article. Further inquiries can be directed to the corresponding authors.
